# Soluble interleukin-2 receptor, intercellular adhesion molecule-1 and interleukin-10 serum levels in patients withelanoma

**DOI:** 10.1054/bjoc.2000.1402

**Published:** 2000-09-04

**Authors:** M D Boyano, M D Garcia-Vázquez, T López-Michelena, J Gardeazabal, J Bilbao, M L Cañavate, A García De Galdeano, R Izu, L Díaz-Ramón, J A Raton, J L Díaz-Pérez

**Affiliations:** Department of Cell Biology and Morphological Sciences, School of Medicine and Dentistry, University of the Basque Country, Leioa, Bizkaia 48940, Spain; Department of Dermatology, Cruces Hospital, Baracaldo, Bizkaia 48903, Spain; Department of Preventive Medicine and Public Health, School of Medicine and Dentistry, University of the Basque Country, Leioa, Bizkaia 48940, Spain

**Keywords:** sIL-2R, sICAM-1, IL-10, biological prognostic factors, melanoma progression

## Abstract

Serum soluble interleukin-2 receptor (sIL-2R), intercellular adhesion molecule-1 (sICAM-1) and interleukin-10 (IL-10) have each been reported as useful markers for melanoma progression. To evaluate the clinical relevance of these three markers, we simultaneously analysed their serum levels in patients with melanoma. A longitudinal study with a 3-year follow-up was performed and different stages of the disease were considered. Mean values of sIL-2R were significantly higher than in normal controls in all stages and correlated with the disease progression. The prognosis of patients with levels > 529 U/ml of sIL-2R was significantly poorer than in patients with sIL-2R levels < 529 U/ml. Levels of sICAM-1 were also elevated in melanoma patients, specially at the time of the metastatic disease. Serum IL-10 levels were more frequently detectable in the patients that developed metastasis during follow-up, and the prognosis of patients with detectable IL-10 levels was significantly poorer than in those patients with IL-10 undetected levels. Statistical analysis based on Logistic and Cox regression models showed that only sex, stage and sIL-2R value are factors significantly associated with metastatic progression. Moreover, high levels of sIL-2R could be a risk factor for malignant progression in melanoma. © 2000 Cancer Research Campaign


*British Journal of Cancer* (2000) **83**(7), 847–852
© 2000 Cancer Research Campaign
doi: 10.1054/ bjoc.2000.1402, available online at http://www.idealibrary.com on IDEAL®

### Soluble interleukin-2 receptor, intercellular adhesion molecule-1 and interleukin-10 serum levels in patients with melanoma

**MD Boyano, MD Garcia-Vázquez, T López-Michelena, J Gardeazabal[1], J Bilbao[2], ML Cañavate, A García De Galdeano, R Izu[1], L Díaz-Ramón[1], JA Raton[1] and JL Díaz-Pérez[1]**

Department of Cell Biology and Morphological Sciences, School of Medicine and Dentistry, University of the Basque Country, Leioa 48940, Bizkaia, Spain; [1]Department of Dermatology, Cruces Hospital, Baracaldo 48903, Bizkaia, Spain; [2]Department of Preventive Medicine and Public Health, School of Medicine and Dentistry, University of the Basque Country, Leioa 48940, Bizkaia, Spain

**Summary** Serum soluble interleukin-2 receptor (sIL-2R), intercellular adhesion molecule-1 (sICAM-1) and interleukin-10 (IL-10) have each been reported as useful markers for melanoma progression. To evaluate the clinical relevance of these three markers, we simultaneously analysed their serum levels in patients with melanoma. A longitudinal study with a 3-year follow-up was performed and different stages of the disease were considered. Mean values of sIL-2R were significantly higher than in normal controls in all stages and correlated with the disease progression. The prognosis of patients with levels > 529 U/ml of sIL-2R was significantly poorer than in patients with sIL-2R levels < 529 U/ml. Levels of sICAM-1 were also elevated in melanoma patients, specially at the time of the metastatic disease. Serum IL-10 levels were more frequently detectable in the patients that developed metastasis during follow-up, and the prognosis of patients with detectable IL-10 levels was significantly poorer than in those patients with IL-10 undetected levels. Statistical analysis based on Logistic and Cox regression models showed that only sex, stage and sIL-2R value are factors significantly associated with metastatic progression. Moreover, high levels of sIL-2R could be a risk factor for malignant progression in melanoma. © 2000 Cancer Research Campaign

**Keywords:** sIL-2R; sICAM-1; IL-10; biological prognostic factors; melanoma progression

Cytokines, secreted either by tumour cells or by host cells, play an important role as mediators and/or regulators of tumour–host interactions leading to the progression of malignant tumours (Kerbel RS, 1992). Indeed, the importance of immunologic parameters for diagnosis, prognosis and therapy in clinical oncology is more evident every day. Several of these immunologic parameters related with the malignant progression of melanoma have been reported. Among them, we and other authors have demonstrated that high levels of soluble Interleukin-2 receptor (sIL-2R) in the serum of patients with melanoma could be a predictive factor of metastatic progression (Fierro et al, 1992; Boyano et al, 1997). Soluble IL-2R is a truncated portion of the alpha chain of the IL-2R that is expressed in the membrane and is able to bind to IL-2 with the same affinity as the form anchored to the membrane (Josimovic-Alasevic et al, 1988). The release of sIL-2R is a phenomenon associated with cellular activation and appears to play an important role in regulating the immune response (Fernandez-Botran R, 1991).

The intercellular adhesion molecule-1 (ICAM-1) is a protein associated with the IL-2R, it is in proximity with the high-affinity IL-2 receptor and interacts physically with it (Burton et al, 1990). ICAM-1 is the counter-receptor for leukocyte function-associated antigen-1 (LFA-1) (Marlin and Springer, 1987; Staunton et al, 1990). The LFA-1/ICAM-1 interaction is important in a number of leukocyte adhesion activities, including the conjugate formation between cytotoxic T lymphocytes (CTL) and their targets (Vanky et al, 1990) and natural killer (NK)- or lymphokine-activated killer (LAK)- mediated cytolysis (Timonen et al, 1988). Together with ICAM-1 tissue expression, the existence of soluble forms of this molecule has been demonstrated in the serum of normal subjects, and serum levels of soluble ICAM-1 are reportedly correlated with the liver metastasis of a variety of tumours and with disease progression in melanoma (Johnson et al, 1989; Tsujisaki et al, 1991; Banks et al, 1993).

Interleukin-10 (IL-10) is a cytokine that contributes to the down-regulation of the immune response. It was described as a cytokine inhibiting several different cell functions of the immune system, including T- and NK-cell and monocyte/macrophage functions (Fiorentino et al, 1991; Taga and Tosako, 1992). IL-10 mRNA is expressed in human melanoma cell lines (Chen et al, 1994), and elevated serum levels of IL-10 in patients with metastatic malignant melanoma have been observed (Dummer et al, 1995). However, IL-10 exhibits antitumoral and antimetastatic activity in some experimental systems (Huang et al, 1996; Landáyi et al, 1998).

The aim of the present study is to evaluate the prognostic impact of the combined detection of sIL-2R, sICAM-1 and IL-10 in the serum of patients throughout several stages of melanoma. For this purpose, patients were followed longitudinally through repeated sIL-2R, sICAM-1 and IL-10 measurements over a three-year follow-up period.

*Received 23 November 1999*
*Revised 7 June 2000*
*Accepted 22 June 2000*

*Correspondence to:* MD Boyano

**Table 1** Clinical data of patients

| | No. of patients in group N[a] | Sex | No. of patients in group F[a] | Sex |
|---|---|---|---|---|
| Melanomas | 109 | 40M/69W | 133 | 40M/93W |
| stage[b] | | | | |
| IA | 28 | 10M/18W | 27 | 8M/19W |
| IB | 28 | 4M/24W | 42 | 8M/34W |
| IIA | 34 | 13M/21W | 52 | 18M/34W |
| IIB | 16 | 10M/6W | 10 | 6M/4W |
| III | 3 | 3M/0W | 2 | 0M/2W |

[a] Group N, patients whose diagnosis and surgery for primary melanoma occurred during the period of time of the study and whose serum samples were tested within the first month after surgery. Group F, patients who had undergone surgery for primary melanoma several years before first serum determinations. [b] Staging system for melanoma is in accordance to the American Joint Committee.

### MATERIALS AND METHODS

### Patients

The study focused on 242 patients with histologically confirmed malignant melanoma (162 women and 80 men, mean age 52 years, range 19–77). The patients were untreated except for primary surgery, and were free of infections as judged by clinical evaluation and absence of raised infectious parameters in the blood. After surgery of the primary tumour, patients go through medical examination every three months during the first two years and after that, every six months until five years of follow-up. After the fifth year, they attend an annual revision up to the tenth year. Those patients who develop metastasis during the follow-up period are newly examined every three months for the following two years. The presence or absence of metastasis was assessed in all patients by physical examination, laboratory and radiological tests. The appearance of metastasis was always confirmed by radiographic examination and/or computed tomography scanning. Disease stages were classified according to the American Joint Committee on Cancer (AJCC). Patients' featured are shown in Table 1. The patients were divided into two groups: patients whose diagnosis and surgery for primary melanoma occurred during the study's period of time and whose first serum samples were tested within the first month after surgery (group N, named after new patients), and patients who had undergone surgery for primary melanoma several years before the beginning of this study (group F, named after follow-up patients). 24 patients developed metastasis during follow-up, 13 patients from group N and 11 patients from group F.

After the first serum determination, new serum samples were tested every 3 or 6 months during follow-up, from June 1994 to December 1997. The control group consisted of 58 healthy donors with comparable sex and age distribution characteristics (38 women and 20 men, mean age 47 years, range 22–57).

Patient and control sera, obtained from venous blood samples, were divided into aliquots and stored at –70°C until use.

### Detection of serum sIL-2R, ICAM-1 and IL-10 levels

Serum sIL-2R, sICAM-1 and sIL-10 concentrations were measured using a sandwich enzyme immunoassay. Commercially available kits were used according to the manufacturers' instructions (sIL-2R kit from Immunotech International, France, Marseilles, sICAM-1 and sIL-10 kits from Boehringer Mannheim, Mannheim, Germany). Serum sIL-2R, sICAM-1 and IL-10 concentrations were expressed in U/ml, ng/ml and pg/ml respectively, and the sensitivity of the kits were 50 U/ml, 3.4 ng/ml and 5 pg/ml respectively. Lower levels of these values were regarded as undetectable.

The normal levels of sIL-2R and sICAM-1 were defined by their measurement in healthy controls. Values were considered elevated when they exceeded the mean of controls plus two standard deviations (mean + 2SD).

### Statistical analysis

Differences in sIL-2R and sICAM-1 levels between groups were tested using parametric methods (Student's *t* test for unpaired samples). Differences in sIL-10 levels between groups were tested using nonparametric methods (Mann-Whitney U test for the two groups) and the Chi-square ($\chi^2$) test. Spearman rank-correlation was performed to describe the correlation between serum levels of sIL-2R, sICAM-1 and sIL-10. A $P$ value below 0.05 was considered statistically significant.

The cumulative survival rates were calculated using the Kaplan-Meier methods, and the statistical significance of differences was determined by using the log-rank test ($P < 0.05$). The Logistic and the Cox proportional hazards regression models were used in order to determine predictive factors linked to metastatic progression. Age, sex, stage, metastatic evolution, disease-free interval, sIL-2R, sICAM-1 and sIL-10 levels were all evaluated. We defined the disease-free interval as the period (of months) between surgery for the primary tumour and the appearance of metastasis. For these analyses, patients presenting less than a 2-year follow-up were excluded. Disease-free patients at the end of follow-up period were considered censored observations.

Statistical analysis was performed using SPSS statistical software.

### RESULTS

### Serum levels of sIL-2R, sICAM-1 and sIL-10 in patients with melanoma sIL-2R levels were independent of the age of the healthy controls ($P = 0.27$) and the melanoma patients ($P = 0.84$) and did not vary between sexes ($P = 0.33$ and $P = 0.50$, respectively). At the first serum determination, a significant increase of sIL-2R levels was found in the melanoma group in comparison with the control subjects (Table 2). Also highly significant differences in sIL-2R levels were observed between the patients that developed metastasis during follow-up and the healthy controls. The mean ± SD values of sIL-2R at the time of the first determination in patients of the groups N and F without metastasis were 496 ± 208 U/ml and 494 ± 259 U/ml, respectively. These values increased to 559 ± 210 U/ml and 566 ± 198 U/ml respectively during the metastatic disease.

Levels higher than 529 U/ml (i.e. mean + 2SD of the mean value of healthy control subjects) were only found in 1 out of 58 controls (1.7%) and 11 out of 96 (11.5%) patients at the time of the primary melanoma. However, at the appearance of metastasis, a 53.8% of group N patients and 54.5% of group F presented high levels of sIL-2R.

On the other hand, when serum sIL-2R levels and stage of the disease were compared, the mean value of IIA+IIB was significantly

 © 2000 Cancer Research Campaign

**Table 2** sIL-2R and sICAM-1 serum levels in melanoma patients

| | | Cases | sIL-2R (U/ml) Mean± SD | sIL-2R > 529 U/ml (%)[a] | sICAM-1 (ng/ml) Mean ± SD | sICAM-1 > 455 ng/ml (%)[b] |
|---|---|---|---|---|---|---|
| Controls | | 58 | 323 ± 103 | 1.7 | 295 ± 80 | 0 |
| Melanomas | | 109 | 393 ± 228 $P < 0.05$ | 11.5 | 342 ± 99 $P < 0.05$ | 15.6 |
| Primary tumour | | 96 | 379 ± 228 *NS* | 11 | 340 ± 99 $P < 0.005$ | 13.5 |
| Metastasis | group N | 13 | 496 ± 208 $P < 0.005$[c] | 23.1[c] | 357 ± 99 $P < 0.05$[c] | 23.1[c] |
| | | | 559 ± 210 $P < 0.005$[d] | 53.8[d] | 413 ± 137 $P < 0.005$[d] | 38.5[d] |
| | group F | 11 | 494 ± 259 $P < 0.005$[c] | 45.5[c] | 390 ± 155 $P < 0.005$[c] | 27.5[c] |
| | | | 566 ± 198 $P < 0.005$[d] | 54.5[d] | 439 ± 133 $P < 0.005$[d] | 45.5[d] |
| Stage | | | | | | |
| IA + IB | | 56 | 377 ± 244 *NS* | 12.7 | 328 ± 97 *NS* | 8.9 |
| IIA + IIB | | 50 | 418 ± 261 $P < 0.01$ | 16 | 359 ± 103 $P < 0.005$ | 30 |
| III | | 3 | 437 ± 115 | 0 | 328 ± 64 | 0 |

[a] Percentage of patients with levels of sIL-2R > 529 U/ml (mean + 2SD with respect to the mean value of controls). [b] Percentage of patients with levels of sICAM-1 > 455 ng/ml (mean + 2SD with respect to the mean value of controls). [c] First serum sIL-2R and sICAM-1 determinations. [d] sIL-2R and sICAM-1 values at the time of the metastasis. Group N, patients whose diagnosis and surgery for primary melanoma occurred during the period of time of the study and whose serum samples were tested within the first month after surgery. Group F, patients who had undergone surgery for primary melanoma several years before the first serum determinations. $P < 0.05$, significant versus controls, NS: not significant.

higher than in controls. Three stage III patients presented a non statistically valuable moderate increase of sIL-2R (437 ± 115 U/ml).

Soluble ICAM-1 and IL-10 levels were also quantified in the N group of melanoma patients. As shown in Table 2, the mean sICAM-1 levels in controls was 295 ± 80 ng/ml. Significant differences were detected in relation to age ($P < 0.01$) but not to sex ($P = 0.34$). In melanoma patients, sICAM-1 value was significantly higher than in controls (342 ± 99 ng/ml) and no sex- or age-related differences were found ($P = 0.36$ and $P = 0.43$, respectively). Also, higher levels were detected in patients of groups N and F that developed metastasis during follow-up than in controls.

Levels higher than 455 ng/ml (mean + 2SD) were observed in 17 out of the 109 (15.6%) melanoma patients and none were found in the controls. Thirteen out of 96 (13.5%) patients with primary melanomas had high levels of sICAM-1. Also 3 out of 13 (23.1%) and 3 out of 11 (27.5%) who belonged to the groups N and F respectively, presented high levels before development of metastasis. When the sICAM-1 determination was performed at the time of the metastatic disease, the percentages raised to 38.5% and 45.5%, respectively. According to the disease stage, patients with stages IIA + IIB had significant higher levels than patients with stages IA + IB. Five out of 56 (12.7%) patients with IA + IB and 15 out of 50 (20%) patients with IIA + IIB stages had sICAM-1 levels higher than 455 ng/ml. Values of sICAM-1 in the three stage III patients were 328 ± 64 ng/ml.

During follow-up, two or more serum determinations were analysed each year. In patients who remained disease-free, no significant differences were found between the sIL-2R and sICAM-1 levels at 12, 24 and 36 months after the first determination (data not shown). However, an increase of sIL-2R serum levels until the time of the metastatic disease was observed in 79% of patients (Fig. 1). Only a 40% presented high levels of sICAM-1.

Survival analysis showed that the prognosis of melanoma patients from Total and F groups with high levels of sIL-2R at the first measure was significantly poorer than that of patients with sIL-2R < 529 U/ml ($P = 0.0143$ and $P = 0.0080$, respectively) Fig. 2A and B). Excluding patients of the group N with less than 2-years follow-up, there was also a significant correlation between high levels of sIL-2R and prognosis ($P = 0.0154$). However, there was no significant correlation between high levels of sICAM-1 and prognosis ($P = 0.6361$) (Fig. 2D).

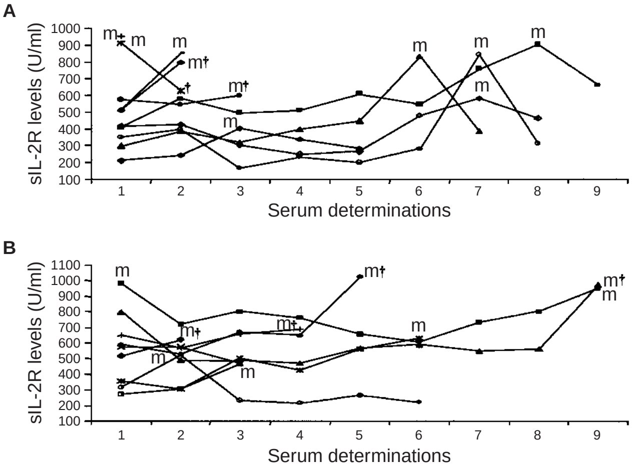

**Figure 1** Individual serum levels of sIL-2R in melanoma patients that developed metastasis during follow-up. (**A**) Group N of patients (n = 10); (**B**) Group F of patients (*n* = 9). m = metastasis; † = death

Serum IL-10 levels did not correlate with age and sex, in both the control ($P = 0.541$ and $P = 0.797$, respectively) and melanoma groups ($P = 0.143$ and $P = 0.364$, respectively). 78 out of 109 (62.3%) melanoma patients and 36 out of 56 (64%) healthy controls showed detectable serum IL-10 concentrations (Table 3). The proportion of patients with detectable IL-10 levels at the time of primary tumour was similar to that of the controls (58%). In the patients that developed metastasis during follow-up, serum IL-10 levels were more frequently detectable (85% in group N and 91% in group F of patients) at the first serum determination. The analysis of IL-10 values by the Chi-square ($\chi^2$) test showed that the ratio of probabilities to detect IL-10 in the serum of patients that developed metastasis during follow-up was 2.35 (1.62 < PR < 3.41) (confidence interval of 95%).

The division of patients into two groups according to the serum IL-10 levels – detectable and non-detectable – revealed that the prognosis of patients with undetectable IL-10 levels was significantly better than those with detectable levels (Log rank = 4.44, $P = 0.0352$) (Fig. 2E). However, at the time of the metastatic disease, and in the patients that remained disease-free after

© 2000 Cancer Research Campaign 

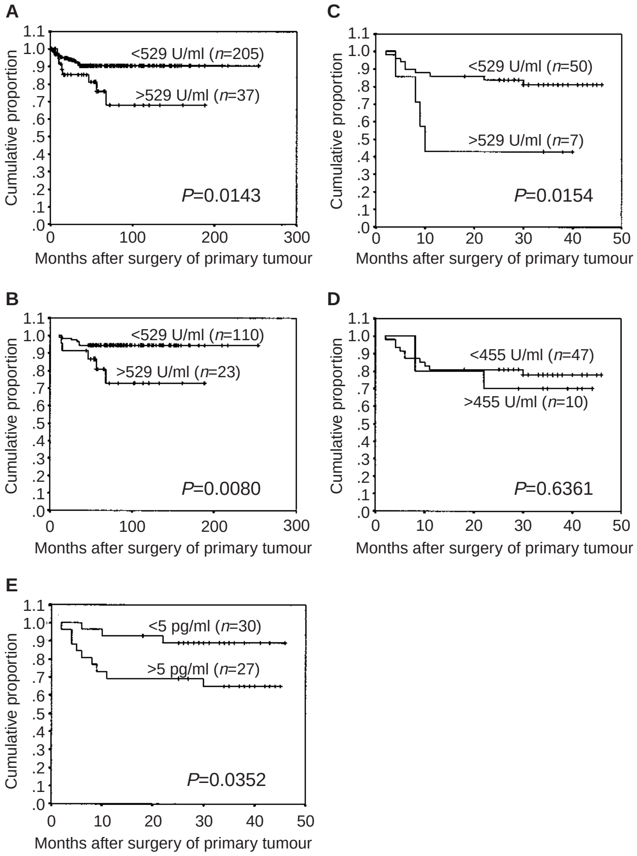

**Figure 2** Disease-free survival curves for melanoma patients in relation to their serum levels of sIL-2R, sICAM-1 and IL-10. (**A**) Total group of patients ($n$ = 242), subdivided according to sIL-2R serum levels; (**B**) Group F of patients ($n$ = 133) subdivided according to sIL-2R serum levels; (**C, D** and **E**) Group N of patients subdivided according to sIL-2R, sICAM-1 and IL-10 serum levels, respectively. Patients with less than 24 months follow-up were excluded. Statistical significance ($P$ < 0.05)

24 months of the first IL-10 measurement, a significant decrease in the percentage of patients with detectable IL-10 levels was observed. Comparison of IL-10 serum levels between controls and melanoma patients performed by the nonparametric Mann-Whitney U test, demonstrated a decrease of IL-10 levels statistically significant in melanoma patients.

We also analysed the possible correlation between serum sIL-2R, sICAM-1 and IL-10 concentrations and found that during follow-up, 12 months after the first serum level determinations, circulating sICAM-1 was negatively correlated with IL-10 serum levels (r = − 0.204, $P$ = 0.047).

Finally, to determine which factors might be predictive of metastatic progression in melanoma, we employed the Logistic and Cox regression models. Only group N of patients met the requirements for analysis with these methods. The factors considered were age, sex, stage, disease-free interval and the serum levels of sIL-2R, sICAM-1 and IL-10. We first used a Logistic proportional hazards regression model to determine the most informative combination of independent factors for prognosis. The results showed that sIL-2R ($P$ = 0.001), sex (male) ($P$ = 0.045) and stage ($P$ = 0.013) were significantly associated with the metastatic progression of melanoma. As in our study we have examined the serum levels at different points in time for the same patients, we used the Cox regression analysis with time dependent covariates and observed that sIL-2R levels could be used as a prognostic marker for the metastatic progression of melanoma ($P$ = 0.002) (Table 4).

### DISCUSSION

This paper describes serial measurements of soluble sIL-2R, sICAM-1 and IL-10 in the serum of patients with melanoma during a three-year follow-up period. In accordance with a previous study, we observed that serum sIL-2R levels are elevated in patients with melanoma in comparison to healthy controls. This increase was significantly higher in the group of patients that

**Table 3** sIL-10 serum levels in melanoma patients during the follow-up

| | | First determination | | After 12 months | | After 24 months | |
|---|---|---|---|---|---|---|---|
| | | Cases (%)[a] | IL-10 ng/ml Mean ± SD (range) | Cases (%)[a] | IL-10 ng/ml Mean ± SD (range) | Cases (%)[a] | IL-10 ng/ml Mean ± SD (range) |
| Controls | | 56 (64) | 7.3 ± 7.5 (0–35) | | | | |
| Melanomas | | 109 (62.3) | 8.2 ± 10 (0–69) | 86 (48.8) | 6.1 ± 9 (0–58) | 47 (25.5) | 2.3 ± 4.5* (0–17) |
| Primary tumours | | 96 (58) | 8.3 ± 11.1 (0–69) | 74 (45) | 5.8 ± 9.7 (0–58) | 44 (28) | 2.3 ± 4.7* (0–17) |
| Metastasis | group N | 13 (85)[b] | 8.0 ± 5.2[b] (0–21) | 12 (75)[b] (0–21) | 7.9 ± 6.6[b] | 5 (20)[b] | 1.6 ± 3.6[b] (0–8) |
| | | (54)[c] | 7.0 ± 8.7[c] (0–27) | | | | |
| | group F | 11 (91)[b] | 14 ± 7.3[b] (0–30) | 9(89) | 10.8 ± 8.0[b] (0–29) | 6 (34)[b] | 3.3 ± 5.2[b] (0–11) |
| | | (54)[c] | 11.0 ± 14.3[c] (0–50) | | | | |
| Stage | | | | | | | |
| IA + IB | | 56 (63) | 7.4 ± 9.1 (0–56) | 42 (50) | 7.4 ± 11 (0–58) | 22 (27) | 2.7 ± 5.0* (0–17) |
| IIA + IIB | | 50 (60) | 8.9 ± 12 (0–69) | 41 (44) | 4.8 ± 7.3 (0–32) | 25 (24) | 2.0 ± 4.2* (0–17) |
| III | | 3 | 6.0 ± 5.3 (0–10) | 2 | 7.5 ± 4.9 (0–11) | 0 | – |

[a] Percentage of melanoma patients with detectable sIL-10. Comparison of serum IL-10 levels between controls and melanoma patients by nonparametric Mann-Whitney U test (*$P$ < 0.05). Group N, patients whose diagnosis and surgery for primary melanoma occurred during the period of time of the study and whose serum samples were tested within the first month after surgery. Group F, patients who had undergone surgery for primary melanoma several years before the first serum determinations. [b] First serum IL-10 determination. [c] IL-10 value at the time of the metastasis.

© 2000 Cancer Research Campaign

**Table 4** Summary of multivariate analysis of independent prognostic markers in melanoma patients by the Logistic and Cox proportional hazards model

| Covariates | (*P* value) |
|---|---|
| Logistic regression analysis | |
| Age | 0.1321 |
| Sex | 0.0451 |
| Stage | 0.0131 |
| sIL-2R | 0.0013 |
| sICAM-1 | 0.5773 |
| sIL-10 | 0.1813 |
| Cox regression analysis | |
| *Time-dependent sIL-2R covariate* | |
| [*(1 < T_Cov < 12)*sIL-2RI + (12 < T_Cov < 24)* sIL-2R12 + (24 < T_Cov < 36)*sIL-2R24*][a] | |
| T_Cov | 0.0026 |
| Stage | 0.0557 |
| Sex | 0.2494 |

[a] sIL-2R1 corresponds to the value of the first serum determination and sIL-2R12 and sIL-2R24 correspond to serum determination after 12 and 24 months follow-up.

developed metastasis during follow-up, and the prognoses of patients with elevated serum levels of sIL-2R were significantly poorer than in those patients with normal levels.

The increase of the sIL-2R levels in the serum may be the result of the immune system activation or could be released directly by the individual tumour since melanoma cells can express the IL-2/IL-2R complex (Alileche et al, 1993; Plaisance et al, 1993; García de Galdeano et al, 1996). In both cases, sIL-2R may compete with the cell-surface IL-2 receptor for binding to IL-2 for IL-2-dependent immune responses (Rubin and Nelson, 1980). Therefore, a large amount of sIL-2R might induce immunosuppression and correlate with poor prognosis of melanoma patients. In our study, we found that serum levels of sIL-2R can serve as an independent prognostic factor predictive of metastasis progression. Measurements of sIL-2R at the time of the primary tumour can be a useful tool to identify melanoma patients with a significant risk to develop metastasis during follow-up. Also, high sIL-2R levels indicate a poor prognosis in patients who had undergone surgery for primary tumour several years before the determination of sIL-2R.

The sICAM-1 is another molecule that appears to be closely correlated with the progression of melanoma (Natali et al, 1990; Harning et al, 1990; Altamonte et al, 1991; Giavazzi et al, 1992; Kageshita et al, 1993). It has been suggested that sICAM-1 may represent a mechanism by which tumour cells escape from the cytotoxicity mediated by immunoeffector cells. Soluble ICAM-1, by competition with the membrane-bound ICAM-1, interferes with the physical interaction of NK and LAK cells with melanoma cells, thus reducing their susceptibility to cytotoxicity (Becker et al, 1991). In our study, serum levels of sICAM-1 in the patients analysed were significantly higher than those of controls. This increase was more evident in the group of patients that developed metastasis during follow-up and, even more, at the time of the metastatic disease.

Similar results were found when IL-10 serum levels were evaluated. At the first determination, serum IL-10 levels were more frequently detected (2.35 times) in the group of patients that developed metastasis during follow-up than in the patients that remained disease-free. The prognosis of patients with detected IL-10 levels was poorer than in those with undetectable IL-10 levels, indicating that detectable levels of IL-10 might contribute to down-regulated anti-tumour response. However, the proportion of patients with detectable serum levels of IL-10 during the period of the metastatic disease was similar to that of the controls.

IL-10 is a pleiotropic cytokine that can exert either immunosuppressive or immunostimulatory effects on a variety of cells types. IL-10 is a potent inhibitor of monocyte/macrophage function (Fiorentino et al, 1991). By contrast, IL-10 can act on B cells to enhance their viability, cell proliferation, Ig secretion and class II MHC expression (Howard et al, 1992). On the other hand, melanoma cells are able to produce IL-10 (Sato et al, 1996), suggesting that IL-10 could contribute to immunosuppression by melanoma (Chen et al, 1994). The results of our study could lead us to think that after the surgery of the primary melanoma, detection of IL-10 in the serum of patients might be reflecting a suppressive status of the immune response. So, high serum levels of IL-10 together with high levels of sIL-2R and sICAM-1 could be identifying patients that might develop metastasis during the follow-up.

Finally, it has been demonstrated that IL-10 is an autocrine growth factor for human melanoma cells and down-regulates the expression of cellular ICAM-1 on melanoma cells (Yue et al, 1997). In this study, a significant negative correlation between IL-10 levels and sICAM-1 levels has been observed during follow-up, after 12 months of the surgery of primary melanoma.

Analysis of the different factors (sex, age, stage, disease-free interval, serum sIL-2R, sICAM-1 and IL-10 levels) that might be correlated with the metastatic progression of melanoma showed that sIL-2R levels, sex and stage were linked to malignant progression, and that sIL-2R has a predictive value for the metastatic progression of melanoma. In contrast to previous studies (Schadendorf et al, 1996), we could not prove that sICAM-1 and IL-10 are statistically associated to the metastatic progression of melanoma. These results may be due to the fact that we did not evaluate patients in advanced stages of melanoma progression.

In conclusion, if progression of melanoma can be attributed to the tumour cells escaping from the immune system, elevated concentrations of sIL-2R, could have an effect as inhibitors of IL-2 activity. Also, sICAM-1's capacity to inhibit MHC-restricted specific T-cell melanoma interactions together with the immunosuppressive effect of IL-10, could contribute as well to the metastatic progression of melanoma.

### ACKNOWLEDGEMENTS

We thank Dr. T. Palomares for helpful discussion. This work was supported by grants from the Health Department of the Government of the Basque Country, NOVARTIS S.A. and the Bilbao Bizkaia Kutxa (BBK). MDGV was supported by a fellowship of the Basque Government.

### REFERENCES

Alileche A, Plaisance S, Han DS, Rubinstein E, Mingari C, Bellomo R, Jasmin C and Azzarone B (1993) Human melanoma cell line M14 secretes a functional interleukin 2. *Oncogene* **8**: 1791–1796

Altamonte M, Colizzi F, Esposito G and Maio M (1991) Circulating intercellular adhesion molecule-1 as a marker of disease progression in cutaneous melanoma. *N Engl J Med* **327**: 959–962

Banks RE, Gearing AJH, Hemingway IK, Norfolk DR, Perren TJ and Selby PJ (1993) Circulating intercellular adhesion molecule-1 (ICAM-1), E-selectin and vascular cell-adhesion molecule-1 (VCAM-1) in human malignancies. *Br Cancer Res* **68**: 122–124

© 2000 Cancer Research Campaign 

Becker JC, Dummer R, Hartmann AA, Burg G and Schmidt RE (1991) Shedding of ICAM-1 from human melanoma cell lines induced by IFN-γ and tumour necrosis factor-α. *J Immunol* **147**: 4398–4401

Boyano MD, García-Vázquez MD, Gardeazabal J, García de Galdeano A, Smith-Zubiaga I, Cañavate ML, Ratón JA, Bilbao I and Díaz-Pérez JL (1997) Serum-soluble IL-2 receptor and IL-6 levels in patients with melanoma. *Oncology* **54**: 400–406

Burton J, Goldman CK, Rao P, Moos M and Waldmann TA (1990) Association of intercellular adhesion molecule 1 with the multichain high-affinity interleukin-2 receptor. *Proc Natl Acad Sci USA* **78**: 7329–7333

Chen Q, Daniel V, Maher DW and Hersey P (1994) Production of IL-10 by melanoma cells: examination of its role in immunosuppression mediated by melanoma. *Int J Cancer* **56**: 755–760

Dummer W, Becker JC, Achwaaf A, Leverkus M, Moll T and Brocker EB (1995) Elevated serum levels of interleukin-10 in patients with metastatic malignant melanoma. *Melanoma Res* **5**: 67–68

Fernandez-Botran R (1991) Soluble cytokine receptors: their role in immuneregulation. *FASEB J* **5**: 2567–2574

Fierro MT, Lisa F, Novelli M, Beretero M and Bernengo MG (1992) Soluble Interleukin-2 receptor, CD4 and CD8 levels in melanoma: A longitudinal study. *Dermatology* **184**: 182–189

Fiorentino DF, Zlotnik A, Mosmann TR, Howard M and O'Garra A (1991a) IL-10 inhibits cytokine production by activated macrophages. *J Immunol* **147**: 3815–3822

Fiorentino DF, Zlotnik A, Vieira P, Mosmann TR, Howard M, Moore KW and O'Garra A (1991b) IL-10 acts on the antigen-presenting cell to inhibit cytokine production by Th1 cells. *J Immunol* **146**: 3444–3451

García de Galdeano A, Boyano MD, Smith-Zubiaga I and Cañavate ML (1996) B16F10 murine melanoma cells express interleukin-2 and a functional interleukin-2 receptor. *Tumor Biol* **17**: 155–167

Giavazzi R, Chirivi RGS, Garafalo A, Rambaldi A, Hemingway I and Pigott R (1992) Soluble intercellular adhesion molecule-1 is released by human melanoma cells and is associated with tumour growth in nude mice. *Cancer Res* **52**: 2628–2630

Harning R, Mainolfi E, Bystryn JC, Henn M, Merluzzi VJ and Rothlein R (1990) Serum levels of circulating intercellular adhesion molecule-1 in human malignant melanoma. *Cancer Res* **51**: 5003–5005

Howard M, O'Garra A, Ishida H, de Waal MR and de Vries J (1992) Biological properties of Interleukin-2. *J Clin Immunol* **12**: 239–247

Huang S, Xie K, Bucana CD, Ullrich SE and Bar-Eli M (1996) Interleukin 10 suppresses tumour growth and metastasis of human melanoma cells: potential inhibition of angiogenesis. *Clin Cancer Res* **2**: 1969–1979

Johnson JP, Stade BG, Holtzmann B, Schwable W and Riethmuller G (1989) De novo expression of intercellular adhesion molecule 1 in melanomas correlates with increased risk of metastasis. *Proc Natl Acad Sci USA* **86**: 641–644

Josimovic-Alasevic O, Herrmann T and Diamantstein T (1988) Demonstrations of two distinct forms of released low-affinity type interleukin-2 receptors. *Eur J Immunol* **18**: 1855–1857

Kageshita T, Yoshii A, Kimura N, Ono t, Tsujisaki M, Imai K and Ferrone S (1993) Clinical relevance of ICAM-1 expression in primary lesions and serum of patients with malignant melanoma. *Cancer Res* **53**: 4927–4932.

Kerbel RS (1992) Expression of multi-cytokine resistance and multigrowth factor independence in advanced stage metastatic cancer. *Am J Pathol* **141**: 519–524

Landáyi A, Nagy JO, Jeney A and Tímar J (1998) Cytokine sensitivity of metastatic human melanoma cells lines- simultaneous inhibition of proliferation and enhancement of gelatinase activity. *Pathology Oncology Res* **2**: 108–114

Marlin SD and Springer TA (1987) Intecellular adhesion molecule-1 (ICAM-1) is a ligand for lymphocyte function-associated antigen-1 (LFA-1). *Cell* **15**: 831–843

Natali P, Nicotra MR, Cavalieri R, Bigotti A, Romano G, Temponi M and Ferrone S (1990) Differential expression of intercellulat adhesion molecule 1 in primary and metastatic melanoma lesions. *Cancer* **50**: 1271–1278

Plaisance S, Rubinstein E, Alileche A, Han DS, Sahraoui Y, Mingari C, Bellomo R, Rimoldi D, Colombo MP and Jasmin C (1993) Human melanoma cells express a functional interleukin-2 receptor. *Int J Cancer* **55**: 164–170

Rubin LA and Nelson DL (1990) The soluble interleukin-2 receptor: Biology, function and clinical application. *Ann Inter Med* **113**: 619–627

Sato T, McCue P, Masuoka K, Salwen S, Lattime EC, Mastrangelo MJ and Berd D (1916) Interleukin 10 production by human melanoma. *Clin Cancer Res* **2**: 1383–1390

Schadendorf D, Diehl S, Zuberbier T, Schadendorf C and Henz BM (1996) Quantitative detection of soluble adhesion molecules in sera of melanoma patients correlates with clinical stage. *Dermatology* **192**: 89–93

Staunton DE, Daustin ML, Erickson HP and Springer TA (1990) The arrangement of the immunoglobulin-like domains of ICAM-1 and the binding sites for LFA-1. *Cell* **61**: 243–254

Taga K and Tosako G (1992) IL-10 inhibits human T cell proliferation and IL-2 production. *J Immunol* **148**: 1143–1147

Timonen T, Patarroyo M and Gahmberg CG (1988) CD11a-c/CD18 and GP84 (LB-2) adhesion molecules on human large granular lymphocytes and their participation in natural killing. *J Immunol* **141**: 1041–1046

Tsujisaki M, Imai K, Hirata H, Hanzawa Y, Masuya J, Nakano T, Sugiyama T, Matsui M, Hinoda Y and Yachi A (1991) Detection of circulating intercellular adhesion molecule-1 antigen in malignant diseases. *Clin Exp Immunol* **85**: 3–8

Vanky F, Wang P, Patarroyo M and Klein E (1990) Expression of the adhesion molecule ICAM-1 and major histocompatibility complex class I antigens on human tumour cells is required for their interaction with autologous lymphocytes *in vitro*. *Cancer Immunol Immunother* **31**: 19–27

Yue FY, Dummer R, Geertsen R, Hofbauer G, Laine E, Manolio S and Burg G (1997) Interleukin-10 is a growth factor for human melanoma cells and down-regulates HLA class-I, HLA class-II and ICAM-1 molecules. *Int J Cancer* **71**: 630–637

 *© 2000 Cancer Research Campaign*
